# High mercury seafood consumption associated with fatigue at specialty medical clinics on Long Island, NY

**DOI:** 10.1016/j.pmedr.2015.09.010

**Published:** 2015-09-25

**Authors:** Shivam Kothari, Danielle Kruse, Roxanne Karimi, Susan Silbernagel, Nurcan Gursoy, Raja Jaber, Heidi Roppelt, Rina Awan, Avram Gold, Jaymie R. Meliker

**Affiliations:** a Program in Public Health, Stony Brook University, NY 11794, United States; b Stony Brook University School of Medicine, NY 11794, United States; c School of Marine and Atmospheric Sciences, Stony Brook University, NY 11794, United States; d Consortium for Inter-Disciplinary Environmental Research, United States; e Division of Allergy and Infectious Diseases, Department of Medicine, University of Washington, Seattle, WA 98195, United States; f Department of Neurology, Stony Brook University School of Medicine, NY 11794, United States; g Department of Preventive Medicine, Stony Brook University School of Medicine, NY 11794, United States; h Department of Rheumatology, Stony Brook University School of Medicine, NY 11794, United States; i Sleep Disorders Center, Department of Medicine, Stony Brook University, NY 11794, United States

**Keywords:** MeHg, Organic mercury = methylmercury, Hg, Mercury, USEPA, United States Environmental Protection Agency, PHQ-9, Patient Health Questionnaire-9, FSS, Fatigue Severity Scale, Fish consumption, Mercury toxicity, Coastal population, Sleep medicine, Mercury exposure

## Abstract

We investigated the association between seafood consumption and symptoms related to potential mercury toxicity in patients presenting to specialty medical clinics at Stony Brook Medical Center on Long Island, New York. We surveyed 118 patients from April–August 2012 about their seafood consumption patterns, specifically how frequently they were eating each type of fish, to assess mercury exposure. We also asked about symptoms associated with mercury toxicity including depression, fatigue, balance difficulties, or tingling around the mouth. Of the 118 adults surveyed, 14 consumed high mercury seafood (tuna steak, marlin, swordfish, or shark) at least weekly. This group was more likely to suffer from fatigue than other patients (p = 0.02). Logistic regression confirmed this association of fatigue with frequent high mercury fish consumption in both unadjusted analysis (OR = 5.53; 95% CI: 1.40–21.90) and analysis adjusted for age, race, sex, income, and clinic type (OR = 7.89; 95% CI: 1.63–38.15). No associations were observed between fish intake and depression, balance difficulties, or tingling around the mouth. Findings suggest that fatigue may be associated with eating high mercury fish but sample size is small. Larger studies are needed to determine whether fish intake patterns or blood mercury tests warrant consideration as part of the clinical work-up in coastal regions.

## Highlights

• We examined seafood consumption and symptoms related to mercury toxicity.
• 118 patients from several specialty medical clinics participated.
• Patients consuming high-mercury seafood were more likely to suffer from fatigue.
• No associations were observed between fish intake and other symptoms.
• Sample size is small; results need to be confirmed in larger study.

## Introduction

Fish consumption has many health benefits, and some Americans are attempting to eat more seafood as a part of a balanced diet (Kris-Etherton et al., 2003, Smith et al., 2009, Blasbalg et al., 2011); however, certain types of fish contain levels of organic mercury (methylmercury, MeHg) that may cause adverse health outcomes (Balshaw et al., 2007, Hightower and Moore, 2003). MeHg in natural waters enters lower trophic level species, bio-accumulates in the fish's body and biomagnifies through the food web, resulting in high concentrations in predatory fish (Morel et al., 1998, Jarup, 2003, Cabana et al., 1994). MeHg is easily absorbed in the human digestive tract (Balshaw et al., 2007, Rice et al., 2014). While the prenatal risks of high MeHg exposure have been well known for decades (Ekino et al., 2007), health outcomes associated with low levels of exposure are less clear. Case reports and small cohort studies in populations of avid seafood consumers indicate a range of potential adverse health effects. These include neurobehavioral (Carta et al., 2003, Yokoo et al., 2003, Masley et al., 2012), neurodevelopmental (Oken et al., 2005, Grandjean et al., 1997), immunotoxic (Council, 2000, Gardner et al., 2010), cardiovascular (Stern, 2005, Goodrich et al., 2013, Choi et al., 2009), and generalized symptoms including sleep disturbance, headache, fatigue, memory loss, muscle and joint pain, peripheral neuropathy, tingling around the mouth, heart rate disturbance, and vision problems (Hightower and Moore, 2003, Silbernagel et al., 2011). These nonspecific symptoms have poorly defined etiology, and the level at which symptoms appear varies across individuals (Gundacker et al., 2010, Koedrith et al., 2013). Nevertheless, levels as low as < 7 μg/L have been reported as associated with symptoms in some adults (Silbernagel et al., 2011) although there are very few studies of risks in the adult population of seafood consumers.

Hg toxicity from seafood may be under-diagnosed (Hightower and Moore, 2003, Silbernagel et al., 2011), possibly due to the non-specific nature of the symptoms, many of which occur from other common diseases. We recently reported that 31–42% of adult avid seafood consumers on Long Island, New York had blood Hg levels above 5.8 μg/L (Karimi et al., 2014), the blood level associated with the USEPA reference dose for MeHg to avoid adverse effects over a lifetime of exposure. The same study found that consumption of shark, swordfish, marlin or tuna steak at least weekly correlates with an increase in blood Hg that places individuals above the USEPA reference dose (Karimi et al., 2014). These findings are consistent with a cross-sectional study of New York City residents that found about 25% of all adults and almost 50% of Asian New Yorkers had blood Hg levels at or above this threshold (McKelvey et al., 2007). Additionally, a previous report suggests that people from coastal regions have higher blood Hg levels due to increased seafood consumption (Mahaffey et al., 2009). None of these studies report on adverse health effects of these elevated blood mercury levels due to increased seafood consumption.

Since the symptoms of Hg toxicity are non-specific and public awareness is low (Ser and Watanabe, 2012, Lin et al., 2014), patients who experience symptoms resulting from high Hg exposure may seek testing or treatment for more common diseases with similar presentations. Additionally, there are currently only case reports correlating high Hg seafood consumption with health outcomes such as fatigue. The intent of our study was to investigate the association between seafood consumption and symptoms potentially related to Hg toxicity among adult patients presenting to several specialty clinics at Stony Brook University and its affiliate patient centers. We chose clinics likely to have patients presenting with Hg toxicity-like symptoms, including Rheumatology, Integrative Medicine, Neurology and Sleep Medicine. The goal of our small study was to determine whether increased consumption of high Hg fish was associated with adverse health outcomes related to low level Hg toxicity.

## Methods

**Fig. 1:**
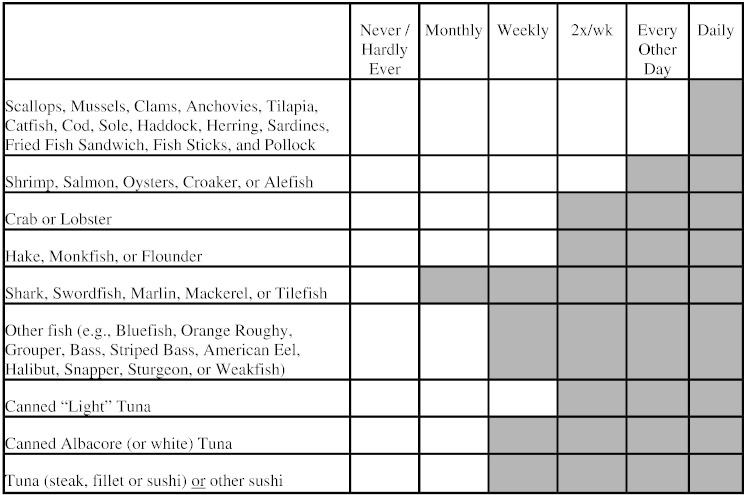
Screening survey used to determine the need for a blood Hg test. We recommended that physicians order a blood Hg test if the response was in the gray shaded region, or if there were a total of six checkmarks in any region. The version that the patients completed did not include a shaded region.

From April through August 2012, patients reporting to Stony Brook Medical Center Rheumatology, Integrative Medicine, Neurology, and Sleep Clinics were invited to participate in our study. Passive recruitment included signs and study flyers available in waiting areas; these patients were enrolled by their physicians during their appointments. Medical students also actively recruited patients waiting to be seen by these specialty physicians one day per week in each clinic for 8 weeks. The study was explained to subjects, and interested patients were asked to sign an informed consent document and complete a brief screening survey about their seafood consumption (Fig. 1).

Responses that were considered “at risk” included at least one check mark in the shaded region of the survey form, which corresponds to higher Hg seafood (Fig. 1) or at least 6 check marks in any region. Fish consumption data and demographic information were obtained from the screening survey instrument. Physicians were asked to report the patient's primary diagnosis.

Patients also completed a health survey that included self-reported data about difficulty balancing, experiencing tingling around the mouth, an assessment for depression—the PHQ-9, and an assessment for fatigue—the Fatigue Severity Scale (FSS) (Table 3). The PHQ-9 is a validated Patient Health Questionnaire with Cronbach's alpha of 0.87 that involves 9 questions that are scored to identify patients on a scale as either having “none” or “mild” to “severe” depression (Kroenke, 2002, Milette et al., 2010). We used this to classify patients as not depressed or depressed, with any patient scoring with at least “moderate” depression being counted as depressed. This is based on recommendations for treatment for patients whose scores reflect at least moderate depression (Kroenke, 2002). The FSS is a validated Fatigue Severity Scale with Cronbach's alpha of 0.93, and has 10 questions that assess fatigue; if patients' average score is 4 or greater, they are considered positive for fatigue, based on the design of this tool (Krupp et al., 1989, Valko et al., 2008). These self-reported health measures were investigated in association with weekly consumption of high Hg fish (tuna steak, swordfish, shark, marlin) (Karimi et al., 2012) using Fisher's exact test. Logistic regression analyses were performed using SPSS investigating fish intake in relation to the different health endpoints, adjusting for age as a continuous variable and categorical variables sex, race (white, other), income in $US (< 25,000; 25,000–70,000; 70,000–110,000; > 110,000), and clinic type. The health endpoints (difficulty balancing, tingling around the mouth, depression and fatigue) that we chose to analyze in these regressions were those that had at least 15 cases. ANOVA was performed using Excel to assess variation in age, income, race and gender across the specialty clinics, as reported in Table 1, and difference in seafood consumption nationwide as compared to our study population, as well as between our specialty clinics, as reported in Table 2.

This research project was approved by Stony Brook University IRB, #219562.

## Results

The mean age of the 118 consenting participants across the four different clinics was 54.7, with a range of 12–90. There were 86 women and 32 men. The population consisted of 75.4% people of Caucasian descent, 9.3% Hispanic descent, 7.6% African American descent, 2.5% Asian descent, and 5.1% of people who identified as “other” (Table 1). Racial demographics and distribution of average household income were similar to that of Suffolk County, NY (Bureau, 2012). Our study population was more heavily represented by females than the county average, 72.9% versus 50.8%. Demographics were similar among the various clinics (Table 1).

Frequent diagnoses gathered from physicians among these patients were sleep apnea, osteoporosis, peripheral neuropathy, lupus, hypothyroidism, tinnitus, amyotrophic lateral sclerosis, osteoarthritis, and anemia. Symptoms included sleep difficulty, leg pain, memory loss, upper and lower extremity numbness, headaches, double vision, and general weakness and fatigue.

Overall, participants in this study ate fish more frequently compared to the National Health and Nutrition Examination Survey results for women (Mahaffey et al., 2009) (Table 2), as 81.4% of subjects ate seafood at least weekly, with similar consumption frequencies among the clinics.

There were 14 patients who reported eating shark, swordfish, marlin, mackerel, tilefish, or tuna steak/tuna sushi at least weekly; these fish are known to be higher in Hg (Karimi et al., 2014). These patients' symptoms were compared with all other subjects (Table 3), and there were no significant differences except for fatigue, (p < .05) where 64% of the patients who consumed high Hg fish experienced fatigue as compared to 31% of all remaining patients.

Logistic regression analyses showed that eating high Hg fish was associated with fatigue in both unadjusted analysis (OR = 5.53; 95% CI: 1.40–21.90) and analysis adjusted for age, sex, race, income, and clinic type (OR = 7.89; 95% CI: 1.63–38.15). Though not statistically significant, 29% of the patients who consumed high Hg fish experienced tingling around the mouth compared with 13% of the remaining patients, yielding unadjusted (OR = 2.57; 95% CI: 0.71–9.33) and adjusted (OR = 3.86; 95% CI: 0.76–19.64) logistic regression results.

## Discussion

This study found a significant association between consumption of seafood high in Hg and fatigue in patients presenting to specialty clinics in a coastal community. Similar to other coastal regions (Mahaffey et al., 2009), 81.6% of participants in our study were eating seafood at least weekly. We identified 14 patients who consumed swordfish, shark, marlin, or tuna steak at least weekly. We chose this categorization of high Hg fish intake based on findings from one of our previous papers in this region; weekly consumption of swordfish, shark, or marlin was associated with an increase in blood Hg of 9.47 μg/L, while weekly consumption of tuna steak was associated with a 6.30 μg/L increase, both of which would place a person over the reference dose (Karimi et al., 2014). A higher proportion of these 14 patients experienced symptoms indicative of Hg toxicity compared to the remainder of the study group (Table 3), although only fatigue showed statistical significance.

Based on our previous findings, it is likely that many of the subjects who consumed tuna steak/tuna sushi, swordfish, shark, mackerel, tilefish, or marlin at least weekly had elevated blood Hg. Unfortunately, we were unable to systematically collect blood Hg results on our patients. We attempted to collect blood Hg on these patients but only received results for 29 out of the 118 patients, and only 1 of the 14 patients who ate high Hg fish weekly, and that patient had blood Hg < 4.0 μg/L. We do not know the blood Hg level of the other 13 patients who reported eating high Hg fish weekly. The patient or health insurance paid for the blood Hg test, and physicians only prescribed blood Hg tests when they felt it would help in the clinical assessment. A future study that includes collection of blood Hg data for all patients would help elucidate the link between symptoms and blood Hg levels.

This study has several important limitations. First, our sample size is small, with seafood consumption and symptoms data for 118 participants, and only 14 participants who ate high Hg fish frequently (Table 3). Our small sample size contributed to the wide confidence intervals in results from our regression analyses. Additionally, we only had blood Hg testing results for a total of 29 patients, and no results for 13 of the 14 patients in the high exposure group, likely due to the fact that the patient or his/her insurance had to pay for the blood Hg testing. Furthermore, our study is limited by self-reported data and lack of a complete clinical picture of the participants. Specifically, the physicians reported the patients' primary diagnoses in a free-response manner so the responses varied dramatically, and we do not have data on medications the patients were taking. Nevertheless, none of the 14 patients who ate high Hg fish weekly had common diagnoses suggesting this may not be a confounding factor. As with any self-report tool, recall error in the screening survey can play a role in under or over reporting. Patients were also asked to report their own symptoms, which introduces additional recall error. In addition, we did not have information on makeup or dental amalgams or other sources of mercury; nor information about menopause or other clinical diagnoses that are associated with fatigue. However, we did adjust for sex, and when we treat age as a categorical variable (< 55 and ≥ 55) in our adjusted regression model, results do not change appreciably (OR = 7.28; 95% CI: 1.52–34.77). Given this set of limitations, this study should be seen as a first step in investigating seafood consumption and Hg-related symptoms. Previously, only case reports were available on the association between consumption of high Hg seafood and symptoms such as fatigue (Hightower and Moore, 2003, Silbernagel et al., 2011). Our results suggest that future larger, more systematic studies that include the general public and collect blood Hg data are needed to better correlate seafood consumption, blood Hg, and symptoms of the coastal population.

## Conclusions

Our study examined seafood consumption rates and symptoms of potential Hg toxicity of individuals presenting to various specialty clinics at Stony Brook Medical Center on Long Island, New York. Overall, there was a greater prevalence of fatigue in those who reported eating high Hg fish at least weekly. Future studies with larger sample sizes that could directly sample blood Hg levels systematically are needed to elucidate the relationship between high Hg seafood consumption, symptoms and blood Hg levels in adults.

## Conflict of interest statement

The authors declare that there are no conflicts of interest.

## Figures and Tables

**Table 1:** Demographics of the participating patients in the specialty clinics at Stony Brook University, Long Island, New York, Summer, 2012.

	Integrative Medicine (n = 41)	Neurology (n = 40)	Rheumatology (n = 24)	Sleep Disorders (n = 13)	Overall (n = 118)	p-value⁎
Age (years) — mean (SD)	57.1 (19.5)	52.3 (16.6)	51.3 (13.2)	57.8 (15.5)	54.7 (16.9)	0.48
12–40	7 (17%)	7 (17%)	4 (17%)	2 (15%)	20 (17%)	
41–55	7 (17%)	16 (40%)	10 (42%)	4 (31%)	37 (31%)	
56–70	14 (34%)	12 (30%)	9 (37%)	6 (46%)	41 (35%)	
71–90	13 (32%)	4 (10%)	1 (4%)	1 (8%)	19 (16%)	
Sex						0.35
Female	31 (76%)	32 (80%)	15 (63%)	8 (62%)	86 (73%)	
Male	10 (24%)	8 (20%)	9 (37%)	5 (38%)	32 (27%)	
Race						0.05
Caucasian	34 (83%)	32 (80%)	12 (50%)	11 (85%)	89 (75%)	
African American	1 (2%)	4 (10%)	3 (12.5%)	1 (8%)	9 (8%)	
Hispanic	2 (5%)	2 (5%)	7 (29%)	0 (0%)	11 (9%)	
Asian American	1 (2%)	0 (0%)	1 (4%)	1 (8%)	3 (3%)	
Other	3 (7%)	2 (5%)	1 (4%)	0 (0%)	6 (5%)	
Income ($US)						0.26
< 25,000	8 (20%)	4 (10%)	6 (25%)	5 (38%)	23 (19%)	
25,000–70,000	8 (20%)	9 (23%)	5 (21%)	3 (23%)	25 (21%)	
70,000–110,000	10 (24%)	13 (33%)	6 (25%)	3 (23%)	32 (27%)	
110,000–200,000	8 (20%)	5 (13%)	4 (17%)	4 (31%)	21 (18%)	
> 200,000	3 (7%)	5 (13%)	0 (0%)	0 (0%)	8 (7%)	

⁎ p-value represents results of ANOVA between the specialty clinics.

**Table 2:** Fish consumption frequency of patients at Stony Brook University, Long Island, New York, Summer 2012.

	Mahaffey et al., 2009 nationwide data for women (n = 5120)⁎	Overall (n = 118)⁎⁎	Integrative Medicine (n = 41)	Neurology (n = 40)	Rheumatology (n = 24)	Sleep Disorders (n = 13)
None/hardly ever	1220 (24%)	4 (3%)	1 (2%)	1 (3%)	2 (8%)	0 (0%)
1–2 times/month	1470 (29%)	18 (15%)	7 (17%)	9 (23%)	2 (8%)	0 (0%)
1–2 times/week	1917 (37%)	44 (37%)	15 (37%)	13 (32%)	8 (33%)	8 (62%)
3 times/week	301 (6%)	23 (19%)	8 (20%)	7 (17%)	6 (25%)	2 (15%)
4 times/week	212 (4%)	29 (25%)	10 (24%)	10 (25%)	6 (25%)	3 (23%)

⁎ ANOVA of the Mahaffey reported fish consumption and our overall numbers, p-value < 0.0001.

⁎⁎ ANOVA between our specialty clinics, p-value = 0.94.

**Table 3:** Symptoms of subjects at Stony Brook University clinics, Long Island, New York, Summer 2012, among those with and without weekly consumption of high Hg fish.

	Overall (n = 118)	> Weekly consumption of high Hg Fish (n = 14)⁎⁎⁎	< Weekly consumption of high Hg Fish (n = 104)	p value⁎
Trouble with balance	54 (46%)	7 (50%)	47 (45%)	0.78
Tingling around the mouth	18 (15%)	4 (29%)	14 (13%)	0.23
At least moderate depression	19 (16%)	3 (21%)	16 (15%)	0.70
Fatigue	41 (35%)	9 (64%)	32 (31%)	0.02⁎⁎

⁎ p values were calculated using Fisher's exact test.

⁎⁎ p < 0.05.

⁎⁎⁎ Weekly consumption of high Hg fish includes tuna steak/tuna sushi, marlin, shark, swordfish, mackerel, or tilefish.
